# The role of peripheral serotonin in SARS‐CoV‐2 infectivity, COVID‐19 treatment and long COVID

**DOI:** 10.1111/imcb.70097

**Published:** 2026-03-08

**Authors:** Daniel W Thorpe, Lauren A Jones, Alyce M Martin, Rosemary A Coleman, Caitlin Allman, Rochelle A Peterson, Damien J Keating

**Affiliations:** ^1^ Gut Sensory Systems Group, Flinders Health and Medical Research Institute, College of Medicine and Public Health, Flinders University Australia

**Keywords:** COVID‐19, gastrointestinal, SARS‐CoV‐2, serotonin, SSRI

## Abstract

Gastrointestinal symptoms have emerged as a common, but underappreciated, cause of morbidity in relation to SARS‐CoV‐2 infection and the COVID‐19 pandemic. This manifests as a range of indications including diarrhea, anorexia, nausea, vomiting and abdominal pain. In addition, the gastrointestinal tract may represent a route of viral entry via the epithelial cell layer lining the gut wall. This route of entry could be a significant component of disease pathogenesis, including effects on the nervous system via the gut‐brain axis. In this review, we provide an assessment of the effects of COVID‐19 on the gastrointestinal system, its involvement in disease severity and potential pathways for viral entry and infection in the gastrointestinal tract. We also examine evidence that gut‐derived serotonin is affected by SARS‐CoV‐2 infection, how this may link to symptoms and disease pathogenesis and the potential link to the efficacy of selective serotonin reuptake inhibitors in reducing COVID‐19 severity.

## Graphical abstract

In this article, we discuss how SARS‐CoV‐2 infects the gastrointestinal tract, that enterochromaffin cells, the source of circulating serotonin, uniquely express viral entry receptors. We examine how this infection disrupts gut‐derived serotonin, contributing to gastrointestinal symptoms and disease severity, and link elevated plasma serotonin to COVID‐19 pathogenesis and suggest a potential therapeutic role for selective serotonin reuptake inhibitors.

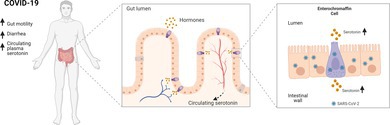

## INTRODUCTION

The coronavirus disease 2019 (COVID‐19) pandemic has had a profound impact on global health, healthcare systems and economies and has resulted in significant loss of life. COVID‐19 arises following infection with the severe acute respiratory syndrome coronavirus 2 (SARS‐CoV‐2) virus and, while multiorgan manifestations exist^1^ {Gavriatopoulou, 2020 #3062}, initial symptoms are largely associated with the respiratory system and significant effects on the gastrointestinal (GI) tract. The GI symptoms associated with COVID‐19 are complex and varied and present a significant new challenge in relation to clinical management of COVID‐19. Additionally, there is significant interest regarding the potential for COVID‐19 to be transmitted via feces and for infection to occur through the GI tract itself. This review will focus on the pathogenic pathways related to acute and chronic GI symptoms identified in relation to COVID‐19 infection, evidence supporting the GI tract as a route of infection, and the future challenges related to this infectious disease, the GI tract and its related physiological pathways.

## GASTROINTESTINAL SYMPTOMS RELATED TO COVID‐19 INFECTION

While COVID‐19 is predominantly a respiratory virus, GI symptoms are reported in 10–60% of hospitalized patients.^2^, ^3^, ^4^ These symptoms include diarrhea, anorexia (loss of appetite), nausea, vomiting, abdominal pain, acid reflux, abdominal distention and bloody stool.^2^, ^5^ The most frequently reported are diarrhea (2–55%), anorexia (10.1–39.7%) and nausea (1.0–27.5%).^6^ While GI symptoms typically occur alongside respiratory symptoms, early reports from China indicated that as many as 23% of patients only experienced GI symptoms.^2^, ^7^, ^8^, ^9^

Patients with severe SARS‐CoV‐2 infection or ICU admission are more likely to experience diarrhea, abdominal pain and anorexia,^8^, ^10^ while those with diarrhea reported an increased prevalence of other common COVID‐19 symptoms such as headaches, fatigue, cough and sputum production.^11^ These patients also experienced longer periods of fever and dyspnea (shortness of breath), took longer to produce a negative throat swab and spent more time in hospital.^11^ However, this finding was not linked with differences in improvement or deterioration of symptoms during follow‐up.^11^ Similarly, patients with GI symptoms were found to experience a longer duration of illness, but that this was not linked to increased severity, as indicated by no difference in intensive care unit (ICU) admissions.^12^ Conversely, while the study by Greco *et al*. on patients in Italy found no difference in disease severity between those with and without GI symptoms, it found that those with GI symptoms spent less time in hospital overall.^13^

GI symptoms appear linked with fecal viral load, a longer period between symptom onset and viral clearance and feature in the presentation of long‐COVID.^9^ A meta‐analysis of findings from 4243 patients with COVID‐19 found that 17.6% of patients had GI symptoms, with viral RNA detected in stool samples from 48.1% of patients, even in stool collected after respiratory samples gave negative test results.^14^ Patients presenting with diarrhea are more likely to produce stool samples that test positive for viral SARS‐CoV‐2 RNA compared to those without diarrhea.^2^, ^9^, ^11^ Similarly, patients with digestive symptoms are more likely to have a longer delay before viral clearance, taking an average of 42 days between onset and clearance compared to 33.5 days.^9^ Long‐COVID has been used to describe a variety of symptoms that persist after COVID‐19 infection, including GI symptoms.^15^ As many as 44% of hospitalized patients have reported experiencing GI symptoms over 90 days from discharge, and up to 8% report GI symptoms after 6 months.^5^, ^15^ The most reported of these were anorexia (24%), nausea (18%), acid reflux (18%) and diarrhea (13%).^5^, ^15^ While these trends do not necessarily translate into COVID severity, they may be indicative of a subgroup of COVID‐19 patients that experience persistent mild GI symptoms of long‐COVID.

## POTENTIAL ROUTES OF SARS‐CoV‐2 ENTRY

The digestive tract is an established route of SARS‐CoV‐2 infection.^16^, ^17^, ^18^ With viable infectious SARS‐CoV‐2 existing in feces from the gastrointestinal tract.^19^ Although gut‐related symptoms alone do not appear to be a predictor of COVID‐19 clinical outcomes or mortality,^20^ the presence of SARS‐CoV‐2 within the gut and degree of GI symptoms is positively correlated with overall prevalence and duration of other COVID‐19 symptoms.^2^, ^3^, ^21^ Infection of human small intestinal organoids with SARS‐CoV and SARS‐CoV‐2 demonstrates that epithelial cells, especially enterocytes, support viral replication.^22^ In similar organoid experiments from human ileum, most gut epithelial cell types were capable of SARS‐CoV‐2 infection, with hormone‐secreting enteroendocrine cells having the greatest proportion of cells infected at 12 h after viral exposure.^23^ Infection via the gut epithelial cell layer may therefore act as a route of infection as well as contribute to the prolonged fecal viral shedding seen in a proportion of infected individuals.^24^

There is now a good understanding of SARS‐CoV‐2‐host interactions, particularly relating to host proteins that are important for viral entry and infection. In this regard angiotensin‐converting enzyme 2 (ACE2), transmembrane serine protease 2 (TMPRSS2), neuropilin‐1 (NRP1), FURIN and Basigin (BSG) have all been implicated.^25^ Conversely, lymphocyte antigen 6 complex, locus E (LY6E), interferon‐induced transmembrane proteins 1 (IFITM1) and 3 (IFITM3), and interferon (IFN) type I receptors (IFNRA1 and IFNRA2) have been associated with a resistance to SARS‐CoV‐2 infection.^25^, ^26^

ACE2 has been identified as the most predominant receptor pathway for multiorgan infection.^27^ Within the GI tract, coexpression of ACE2 and TMPRSS2 facilitates infection of differentiated enterocytes in the small and large intestine.^17^, ^22^ Selective expression of only TMPRSS2 or another key SARS‐CoV‐2 target receptor, neuropilin‐1 (NRP1), is associated with low to no viral infection, indicating that cellular entry requires ACE2 co‐expression.^28^, ^29^ Co‐expression of two or all these facilitatory proteins results in increasing viral loads within cells, highlighted by SARS‐CoV‐2 infection in lung cells enriched in NRP1, TMPRSS2 and the protease enzyme FURIN, compared to noninfected bystander cells^28^ {Cantuti‐Castelvetri, 2020 #3074}. Recently, a suite of SARS‐CoV‐2 targets involved in viral docking, processing and viral defense such as ACE2, TMPRSSR and BSG have been identified as potential mechanisms of viral entry into the brain to cause downstream neurological manifestations.^25^, ^30^

## EXPRESSION OF SARS‐CoV‐2 RECEPTORS AND RELATED PROTEINS IN GUT EPITHELIAL CELLS

For COVID‐19 to have direct effects on the GI tract, we hypothesized that receptors for the virus are intrinsically expressed within the mucosal layer of cells lining the gut wall. To identify the expression pattern of SARS‐CoV‐2 receptors within different cell types of the GI tract, we explored a single‐cell RNA‐seq database for mouse small intestinal mucosa.^31^ Our analysis focused on the expression of genes encoding proteins known or proposed to facilitate COVID‐19 efficient cell entry: ACE2, BSG and NRP1,^29^ associated proteins involved in intracellular trafficking and breakdown: TMPRSS2, TMPRSS11A, TMPRSS11B, FURIN, CTSB and CTSL and proteins linked with viral defence: LY6E, IFITM1‐3 and IFNAR1‐2. We found that all corresponding genes are expressed within the intestinal epithelium^32^ but that the SARS‐CoV‐2 receptor, NRP1, is only expressed in serotonin‐producing enterochromaffin (EC) cells. NRP1 protein expression is highly colocalized in the gastrointestinal wall in cells that express chromogranin‐A (CgA), a marker of EC cells.^33^

## PERIPHERAL SEROTONIN IN COVID‐19

EC cell‐derived serotonin (5‐hydroxytryptamine, 5‐HT) is by far the major source of peripheral, nonbrain serotonin^34^ and coordinates an array of nonneuronal roles including regulation of fat mass^35^ and blood glucose levels^36^ as well as coordinating signals from the gut microbiome to modify host physiology.^37^, ^38^ Gut‐derived serotonin plays a key role in GI function, including gut motility^39^ for which abnormalities in serotonin levels are associated with various gut disorders, including irritable bowel syndrome^40^ and gastroparesis.^41^ EC cell serotonin is modulatory of gut transit, with a constipation phenotype occurring in models lacking EC cell‐derived serotonin,^41^, ^42^, ^43^ and an excess known to cause diarrhea.^44^ A considerable number of COVID‐19 patients report experiencing GI symptoms, with diarrhea being the most common.^2^, ^45^ Circulating plasma serotonin is significantly higher in patients with COVID‐19 compared to healthy patients.^46^, ^47^ Furthermore, plasma serotonin levels correlate strongly with increasing COVID‐19 symptom severity and is significantly higher in COVID‐19 patients with diarrhea compared to those without diarrhea.^46^

## PERIPHERAL SEROTONIN AND IMMUNE RESPONSE

The expression of SARS‐CoV‐2 receptors by EC cells highlights a potential mechanistic pathway not only underlying GI symptomology, but also increased severity of COVID‐19 through its role in the innate and adaptive immune response. The roles of gut‐derived serotonin and serotonin receptors as activators of immune responses and inflammation are well characterized.^48^ Innate immune cells responding to pathogens or infection, such as eosinophils, mast cells, natural killer cells, monocytes, macrophages and dendritic cells, all have the capacity to respond to circulating serotonin through serotonin receptors, whereas monocytes, macrophages, mast cells and dendritic cells can uptake and store serotonin via SERT.^48^ Exogenous serotonin increases mast cell accumulation via 5‐HT_1A_ receptors and uptake through SERT.^49^ Mast cells release pro‐inflammatory mediators such as histamine, proteases, cytokines and chemokines and facilitate the recruitment of other serotonin‐responsive immune cells, such as eosinophils, natural killer cells, neutrophils and adaptive immune cells (dendritic cells and T cells).^50^ Circulating serotonin is thought to contribute significantly to eosinophil infiltration, mediated via 5‐HT_2A_ receptors.^51^ Serotonin increases macrophage production of pro‐inflammatory cytokines, such as interleukin‐1 and interleukin‐6, and enhances their phagocytic capacity.^48^, ^49^ Additionally, serotonin promotes chemotaxis of monocytes to sites of inflammation by working through serotonin receptors expressed on alveolar macrophages.^50^ The relationship between serotonin and inflammation is complex, as several pro‐inflammatory cytokines and immune mediators, including interleukin‐1β and interleukin‐13 also appear to increase EC cell serotonin release.^51^, ^52^, ^53^

Release of several pro‐inflammatory cytokines by mature dendritic cells is enhanced in the presence of serotonin, through activation of 5‐HT_4_ and 5‐HT_7_ receptors.^54^ This leads to the accumulation of immature cells at the site of inflammation and the secretion of inflammatory cytokines that influence downstream T‐cell signaling. As such, serotonin signaling to dendritic cells can have longer‐term consequences for immune function and inflammation, due to their ability to signal cells in the adaptive immune response. Adaptive immunity involves the destruction of invading pathogens through recognition of specific antigens by T and B cells.^55^ Immunological staining of primate intestinal mucosa shows serotonin containing cells are in very close proximity to, and in some cases in direct contact with both T cell and B cells,^56^ suggesting a close relationship between these cell types may exist. Immune cells express a number of serotonin receptors, with 5‐HT_7_ expressed in naïve cells and 5‐HT_1A_ and 5‐HT_2A_ in activated T cells.^57^, ^58^ Similar to T cells, adaptive immune B cells responsible for antigen presentation and antibody production also contain 5‐HT_1A_ receptors, which are upregulated following cell activation.^58^ Expression of SERT has also been found on B cells, indicating uptake and potential storage of serotonin may play a role in modulating B‐cell activity.^59^

This is also demonstrated in mouse models of colitis and intestinal inflammation, with IL‐13 mediating an increase in EC cell number and mucosal 5‐HT content, which is prevented in mice lacking IL‐13 cytokine production and increased with exogenous IL‐13 administration.^51^, ^52^ This was also associated with an increase in infiltrating macrophages, consistent with the activity of serotonin on immune cells. Gut‐derived serotonin worsens intestinal inflammation in animal models of gut inflammatory disorders^60^ due to its effects on increasing macrophage infiltration and production of proinflammatory cytokines through a process dependent on nuclear factor κB signaling in immune cells.^48^ The association between serotonin and inflammation is not limited to the gut, however, with alterations in serotonin levels having also been reported in allergic airway inflammation and rheumatoid arthritis patients.^55^ The role of EC‐cell serotonin in modulating the immune system and peripheral inflammation is evidently complex, with serotonin both contributing to the immune defense system while also being increased by inflammatory cytokines. The latter may be a positive driver to further increase the activity of several immune cells as a means of overcoming infection; however, this may also contribute significantly to diseases associated with increased inflammation, as a heightened response to IL‐1β is seen in EC cells derived from patients with Crohn's disease.^53^

## SELECTIVE SEROTONIN REUPTAKE INHIBITORS AND COVID‐19 TREATMENT

Several recent studies indicate that the use of selective serotonin reuptake inhibitors (SSRIs), normally prescribed to treat mental health conditions such as depression, is highly effective in mitigating COVID‐19 infection *in vitro* ^61^, ^62^ and reducing COVID‐19 severity in humans.^63^, ^64^, ^65^, ^66^ Such drugs act to inhibit the function of the serotonin‐reuptake transporter (SERT) protein. Two such studies demonstrated the ability for SSRIs to either prevent or reduce infection with SARS‐CoV‐2 *in vitro*, using human nasal epithelial cells^61^ and human lung tissue.^62^ Furthermore, a 2020 double‐blind, placebo‐controlled, randomized clinical trial found that fluvoxamine, a common SSRI, significantly reduced clinical deterioration of patients with COVID‐19 compared to placebo.^65^ Clinical deterioration, described as dyspnea, hospitalization, or a decrease in oxygen saturation, occurred in 0 of 80 patients in the fluvoxamine group and 6 in 72 (8.3%) patients in the placebo group. In addition, the placebo group reported more GI symptoms (such as nausea and vomiting) compared with those in the fluvoxamine group.^65^ Similar results were subsequently reported, in which a 2021 randomized, placebo‐controlled, adaptive platform trial found that fluvoxamine treatment resulted in an absolute risk reduction of 5%, and a 32% relative risk reduction of hospitalization due to COVID‐19.^63^ Other observational retrospective studies in 2020 also indicated an association between SSRIs (including escitalopram, fluoxetine and paroxetine and the SNRI venlafaxine) use and a reduction in both intubation requirement and mortality among hospitalized COVID‐19 patients.^64^, ^66^, ^67^, ^68^

A open‐label, 2021 to 2022, randomized, outpatient, controlled trial assessed whether SSRI fluvoxamine in combination with bromhexine, cyproheptadine (antagonist of 5‐hydroxytryptamine) and niclosamid within 48 h of symptom onset of 1900 participates (995 participants completed the trial) would improve clinical deterioration outcomes by 9, 14 or 28 days.^69^ Fluvoxamine, fluvoxamine and bromhexine, and fluvoxamine and cyproheptadine had significantly less clinical deterioration at all time points than standard care participants.^69^ With combination treatments resulting in significantly decreased viral loads at day 5 of treatment, decreased levels of serum cytokines and lower incidence of long COVID‐19 symptoms.^69^ This suggests that SSRI treatment for COVID‐19 may have significant benefits in terms of clinical outcomes. In this regard, a recent meta‐analysis of randomized controlled trials and observational studies found 100 mg twice daily of fluvoxamine was associated with reduced mortality, hospitalization and composite of hospitalization/emergency room visits, while a lower dose of 50 mg twice daily of fluvoxamine did not.^70^ This was consistent with a 2021 to 2022 randomized clinical trial of 1288 outpatients during the Delta and Omicron variants treated with 50 mg of fluvoxamine twice daily for 10 days, compared with placebo, which found no shortened symptom duration.^71^

However, this was met with debate due to the confounding factors such as differences in the major mutated strains at different times, baseline risk of population progression and vaccination rates.^72^ The original authors subsequently agreed that factors beyond differences in fluvoxamine dose should be considered when interpreting their results and that further investigations, including the Fluvoxamine 100 arm of the ACTIV‐6 platform trial would shed light on these issues.^73^ Indeed, that randomized clinical trial in 2022–2023, of 1175 US participants demonstrated that Fluvoxamine, 100 mg twice daily, does not shorten the duration of symptoms in outpatient adults with mild to moderate COVID‐19.^74^ Therefore, in adequately vaccinated populations, there appears minimal evidence that SSRIs are beneficial for patient mortality in COVID‐19.

Vaccinated populations may have a different immunological response, or a decrease in disease severity compared to unvaccinated populations, which may decrease the beneficial effect of SSRIs during COVID‐19 infection.

Variance in the immunological response to COVID‐19 infection may influence the potential benefits of SSRI treatment. A single‐cell transcriptomic and proteomic study during the SARS‐CoV‐2 Delta wave found that vaccinated individuals displayed a less activated immune profile, with reduced monocyte migration pathways and diminished NK cell proliferation.^75^ Increased monocyte migration has been associated with greater disease severity in hospitalized COVID‐19 patients,^76^ suggesting that both vaccination and SSRIs (citalopram, fluvoxamine and fluoxetine) which reduce VCAM‐1 and ICAM‐1, key adhesion molecules for monocyte trafficking^77^ may converge to limit monocyte‐driven pathology.

However, NK cell activity appears to differ. Single‐cell RNA sequencing of NK cells from COVID‐19 patients revealed that enhanced NK cell activation and proliferation correlated with more severe disease.^78^ By contrast, clinical studies of patients with major depressive disorder have shown that 4 week treatment with SSRIs such as escitalopram and fluoxetine increases NK cell activation without affecting NK cell numbers.^79^, ^80^ This suggests that while SSRIs may dampen monocyte migration, their capacity to enhance NK cell activity could instead worsen outcomes in COVID‐19.

Overall, vaccination has substantially reduced COVID‐19 severity, hospitalizations and deaths.^81^, ^82^ As baseline risk decreases with widespread vaccination, the potential therapeutic benefit of SSRIs during infection may have been further diminished.

## LONG COVID‐19 AND SEROTONIN

A recent study identified a link between lower plasma serotonin and Long‐COVID and provides this as an explanation for multiple symptoms including hypercoagulation and cognitive decline.^83^ However, there is debate regarding some of the measurements linking serotonin to long COVID with Anderson *et al*. insisting that the plasma serotonin levels were much higher than expected for platelet free plasma, potentially invalidating the insight of lower plasma serotonin in long COVID.^84^ While the findings from this study remain to be replicated some interesting questions arise. SSRI usage would be expected to be associated with a reduction in plasma serotonin levels, given absence of plasma serotonin is observed in SERT KO mice and rats.^85^, ^86^ This raises the interesting question of whether long COVID is more prevalent in individuals consuming SSRIs. Indeed, this has been addressed in a small study of 95 patients with post‐COVID syndrome in which researchers investigated the efficacy of SSRIs on neurobiological mechanisms.^87^ They reported that all patients experienced improved well‐being and reduced symptoms, with approximately two‐thirds exhibiting a good to strong response to SSRIs,^87^ providing some preliminary evidence of SSRIs' efficacy in treating post‐COVID syndrome. Interestingly, a retrospective analysis of real‐world clinical data from the National COVID Cohort Collaborative explored the potential of SSRIs with sigma‐1 receptor agonist activity to lower the risk of long COVID.^88^ Such receptor activation may provide a mechanism for the anti‐inflammatory effects observed with SSRIs. Thus, SSRIs with and without reported agonist activity at the sigma‐1 receptor were associated with a significant decrease in the risk of Postacute sequelae of COVID‐19.^88^ Such data provide a significant issue in aligning the proposed efficacy of SSRIs in benefitting long COVID, with the proposed observations that low plasma serotonin, which SSRIs should exacerbate, is a significant factor in worsening long COVID frequency and outcomes. We determine that either one of these outcomes is not correct, or that the observed effects do not occur at the level of plasma serotonin levels, but at a more nuanced cell or tissue‐specific role that peripheral serotonin is playing in long COVID. This may include in immune cells, specific brain regions affected via the gut–brain axis or within platelets to alter thrombotic changes in this disorder. Importantly, heterogeneity in how long COVID symptoms were defined and measured across these studies further complicates interpretation of these results.

## CONCLUSIONS

Gastrointestinal symptoms are a well‐characterized component of COVID‐19 and diarrhea is the most common GI symptom in patients with COVID‐19. In addition, circulating levels of serotonin are increased in COVID‐19 and correlate with gastrointestinal symptoms such as diarrhea. All circulating serotonin is produced by EC cells in the gut epithelium and EC cells are the only cell type in the gut wall to express all three receptors for the SARS‐CoV‐2 virus. Peripheral serotonin has well‐characterized effects on the immune system and immune responses, and early data suggested that SSRIs may be efficacious in treating the symptoms and progression of COVID‐19. However, it now appears that larger studies do not support this proposition, especially in those immunized to the virus. While gut serotonin is also implicated in long COVID, significant debate remains on this matter.

## CONFLICT OF INTEREST

The authors have no conflicts of interest to disclose.

## AUTHOR CONTRIBUTIONS

**Daniel W Thorpe**: Writing – original draft. **Lauren A Jones:** Writing – original draft; conceptualization; writing – review and editing. **Alyce M Martin:** Writing – original draft; writing – review and editing; conceptualization. **Rosemary A Coleman:** Writing – original draft. **Caitlin Allman:** Writing – original draft. **Rochelle A Peterson:** Writing – original draft. **Damien J Keating:** Writing – original draft.

## Data Availability

Data sharing is not applicable to this article as no datasets were generated or analyzed during the current study.
